# Near-field fault slip of the 2016 Vettore M_w_ 6.6 earthquake (Central Italy) measured using low-cost GNSS

**DOI:** 10.1038/s41598-017-04917-w

**Published:** 2017-07-04

**Authors:** Maxwell W. Wilkinson, Ken J. W. McCaffrey, Richard R. Jones, Gerald P. Roberts, Robert E. Holdsworth, Laura C. Gregory, Richard J. Walters, Luke Wedmore, Huw Goodall, Francesco Iezzi

**Affiliations:** 1 Geospatial Research Ltd, Department of Earth Sciences, Durham University, Durham, DH1 3LE UK; 2 Department of Earth Sciences, Durham University, Durham, DH1 3LE UK; 3 Department of Earth and Planetary Sciences, Birkbeck, University of London, London, WC1E 7HX UK; 4 School of Earth and Environment, University of Leeds, Leeds, LS2 9JT UK; 5 COMET, Department of Earth Sciences, Durham University, Durham, DH1 3LE UK

## Abstract

The temporal evolution of slip on surface ruptures during an earthquake is important for assessing fault displacement, defining seismic hazard and for predicting ground motion. However, measurements of near-field surface displacement at high temporal resolution are elusive. We present a novel record of near-field co-seismic displacement, measured with 1-second temporal resolution during the 30^th^ October 2016 M_w_ 6.6 Vettore earthquake (Central Italy), using low-cost Global Navigation Satellite System (GNSS) receivers located in the footwall and hangingwall of the Mt. Vettore - Mt. Bove fault system, close to new surface ruptures. We observe a clear temporal and spatial link between our near-field record and InSAR, far-field GPS data, regional measurements from the Italian Strong Motion and National Seismic networks, and field measurements of surface ruptures. Comparison of these datasets illustrates that the observed surface ruptures are the propagation of slip from depth on a surface rupturing (i.e. capable) fault array, as a direct and immediate response to the 30^th^ October earthquake. Large near-field displacement ceased within 6–8 seconds of the origin time, implying that shaking induced gravitational processes were not the primary driving mechanism. We demonstrate that low-cost GNSS is an accurate monitoring tool when installed as custom-made, short-baseline networks.

## Introduction

**Figure 1:**
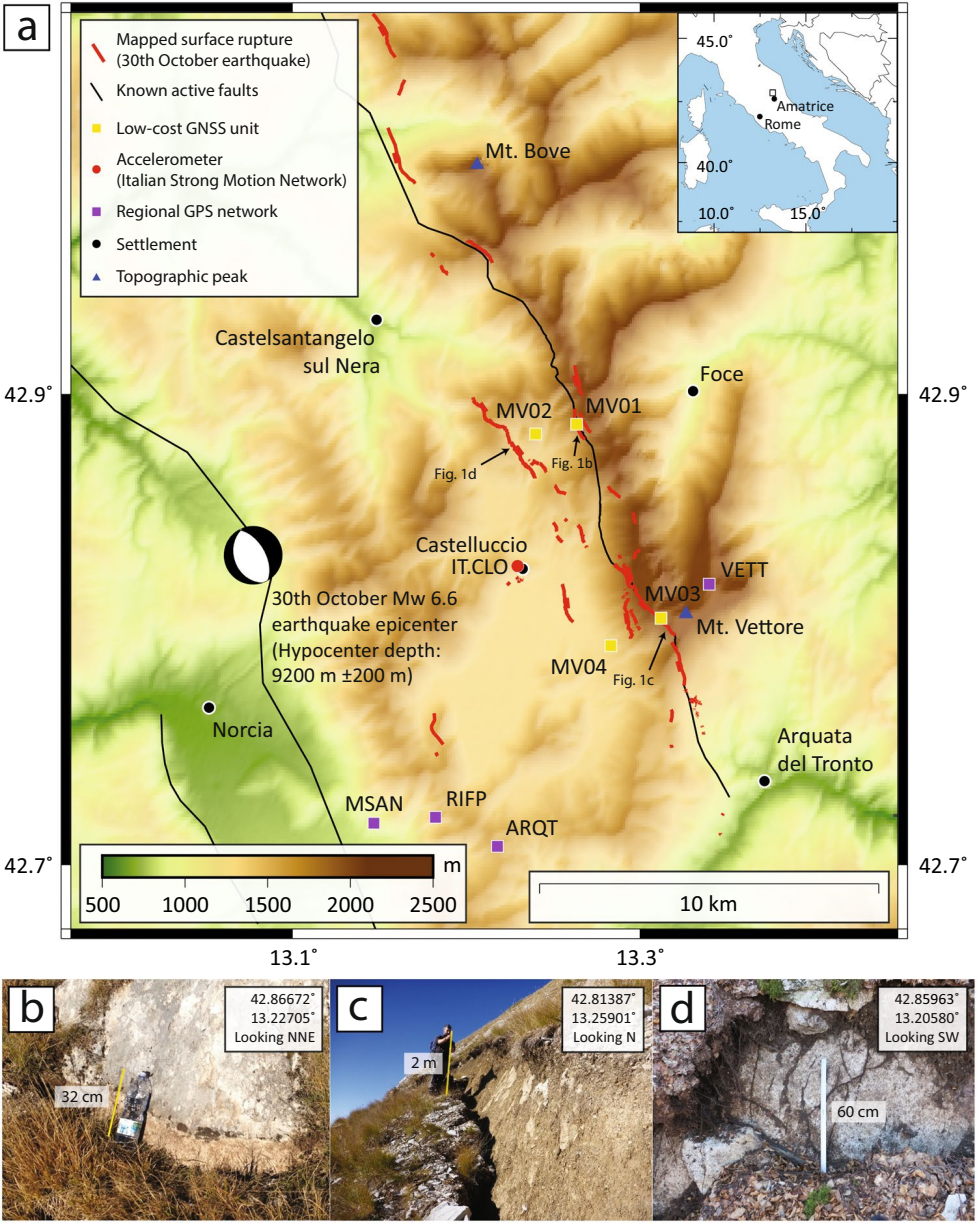
(**a**) Location map of 6.6 Mw October 30th Vettore earthquake. Red lines are surface ruptures from this event mapped by the EMERGEO working group using ground observations. MV01, MV02, MV03 and MV04 are GNSS units used in this study. Additional GNSS stations operated by other researchers are: VETT (INGV – IGM network benchmark), ARQT (INGV, RING network), RIFP and MSAN (INGV – CaGeoNet network). IT.CLO is a strong motion station situated near the village of Castelluccio. The location of the earthquake epicentre was retrieved from http://cnt.rm.ingv.it/en/event/8863681 ^5^. Map coordinates are latitude and longitude decimal degrees of the geographic co-ordinate system WGS84. Topographic elevation is based on 90 m SRTM data^24^. Map generated using GMT software v. 4.5.15 (http://www.soest.hawaii.edu/gmt/)^25^. (**b**) Mt. Vettore fault close to MV01. (**c**) Mt. Vettore fault close to MV03. (**d**) Antithetic fault SW of MV01-MV02 baseline.

Improved measurements of co-seismic fault slip and surface rupture are critical to our understanding of earthquake processes and the design of major infrastructure^1–3^. The 30^th^ October M_w_ 6.6^4^/M_w_ 6.5^5^ earthquake was the largest in Italy for 36 years and forms part of a recent sequence of earthquakes in the central Italian Apennines that includes the M_w_ 6.2^4^/M_w_ 6.0^5^ Amatrice earthquake^6–8^ of 24th August 2016 with 299 fatalities, the M_w_ 5.4^4^ and M_w_ 6.1^4^/M_w_ 5.9^5^ earthquakes of 26th October^9^ and the M_w_ 5.3^4^, M_w_ 5.7^4^, M_w_ 5.6^4^ and M_w_ 5.2^4^ earthquakes of 18^th^ January 2017 that caused 34 fatalities. During the 30^th^ October earthquake ground motions up to 547 gal were recorded at a strong motion station in Accumoli, and shaking of magnitude five on the Mercalli Cancani Sieberg (MCS) scale was felt up to 75 km from the epicentre^10^. Regional GPS stations^10^ measured horizontal co-seismic displacements of 38.3 cm north-east (station VETT, in the footwall) and 26 cm south-west (MSAN, hangingwall). These same stations measured absolute vertical co-seismic displacements of 5.5 cm uplift (VETT, footwall) and 44.6 cm of subsidence (ARQT, hangingwall). The Time Domain Moment Tensor focal mechanism^11^ was extensional, with a strike of 151° and dip of 47° south-west^10^. A maximum slip of 2.5 meters at 4–6 km depth has been inferred^10^. Least squares inversion of the strong motion-derived source model defined a rise time of 2.1 seconds, rupture velocity of 2.5 km/s and total rupture duration of 8 seconds^10^. Inversion of accelerometer data suggest >80 cm of inferred slip at the intersection of the fault and the ground surface, which corresponds well with offsets we measured directly from outcrop in the two days following the earthquake. Surface ruptures within a zone of minimum length of 15 km along the Mt Vettore - Mt. Bove Fault System were mapped remotely by helicopter the day after the earthquake^10^ and in the field by our group, and have helped to identify a complex network comprising of three different west-dipping synthetic fault splays and two antithetic structures (Fig. 1). Preliminary InSAR measurements spanning the 26^th^ October and 30^th^ October earthquakes using Sentinel-1 data of 26 October – 1 November reveal low far-field deformation gradients (1–5 cm/km), with higher gradients up to 30 cm/km observed closer to the Mt Vettore - Mt. Bove Fault System^10^.

## GNSS results from the 30^th^ October earthquake

We recorded positional data using custom-made GNSS units that are designed around a low-cost U-blox NEO-M8T GNSS receiver chip, and built to be compact, robust, weatherproof, and power-efficient. The GNSS chips allow GNSS constellations to be tracked using a single frequency over the L1 band and provide observation data of pseudo-range and carrier phase at rates of up to 10 Hz. These single-frequency observations allow the position of one receiver relative to another to be continually resolved with centimetre spatial accuracy for baselines up to approximately 10 km^12, 13^. The temporal accuracy is sub-microsecond^14^, as GNSS is fundamentally dependant on very precise timing. The GNSS units were assembled with the purpose to monitor surface deformation resulting from earthquakes, but the underlying technology could be applied to many potential monitoring applications within the geosciences, such as volcanic deformation, mass wasting processes, glacial motion or anthropogenic ground movements.

**Figure 2:**
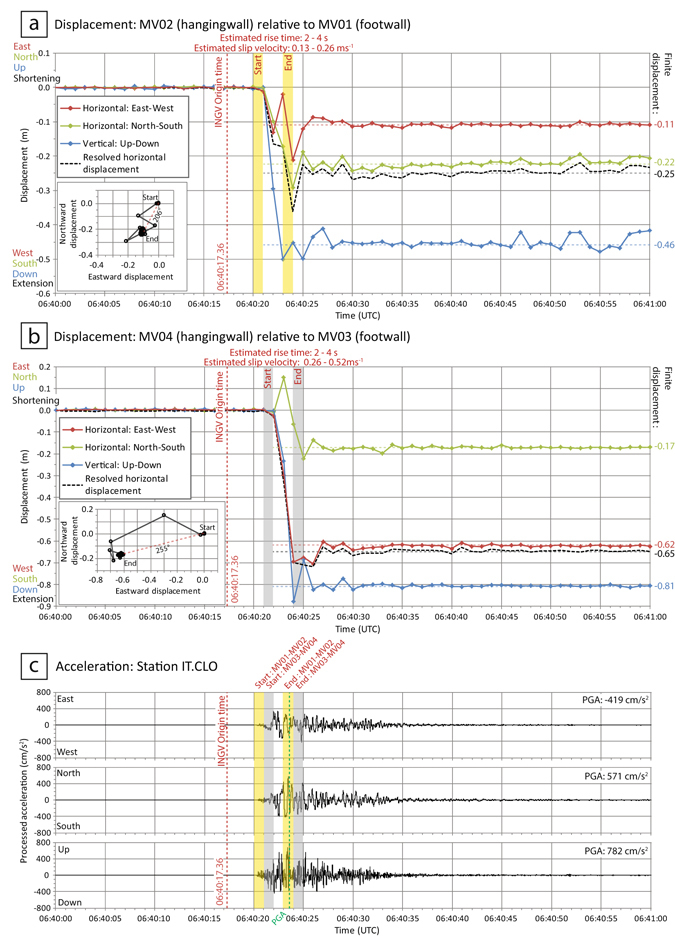
(**a,b**) Time-series of three component relative displacement recorded by GNSS receivers (06:40:00–06:41:00 UTC; see Fig. 1a for locations). Data points represent calculated positions of the GNSS receiver in the hangingwall relative to its corresponding unit in the footwall, at 1 Hz. The inset plot shows the ground track with time on a horizontal plane. Horizontal dashed lines represent the finite co-seismic displacement of each component estimated from the temporal record. Vertical dashed lines represent key times in the sequence. For each GNSS pair, the start and end of co-seismic displacement is estimated as the time when all three components of displacement first reach their finite co-seismic displacement values. Horizontal and vertical error bars are included but are too small to see. (**c**) Three-component acceleration with time for station IT.CLO of the Italian Strong Motion Network^15^. See Fig. 1a for location of IT.CLO.

Following the 24^th^ August Amatrice earthquake we deployed four GNSS units along the Mt. Vettore fault as two footwall-hangingwall pairs, to create two baselines with lengths of 1,286 m and 1,870 m (Fig. 1, Table 1). The along-strike separation of these two baselines is 6.2 km. To eliminate the potential for gravitationally-induced effects in the displacement signal, the locations of the GNSS units were chosen on stable bedrock. The two hangingwall units, MV02 and MV04, are located on a hill within the basin below MV01, and on the Castelluccio plain, respectively. All units recorded positional data at 1 Hz continually to a data card from the time they were installed; there is no triggering mechanism to capture particular seismic events. We deployed the units with the intent to measure the near-field post-seismic deformation of the Mt. Vettore fault following the Amatrice earthquake; by chance, both our baselines were located on a section of the Mt. Vettore fault which subsequently ruptured during the 30^th^ October earthquake. The duration of the data presented here is 1 minute, starting from 06:40:00 UTC, that covers 18 seconds preceding the earthquake and 42 seconds following it (Fig. 2a,b, Supplementary Table S1). The data describe the displacement of the hangingwall GNSS unit relative to the corresponding footwall unit as three components: horizontal east-west, horizontal north-south, and vertical up-down. The resolved horizontal displacement (“horizontal throw”) is derived from the two horizontal components, and corresponds to the incremental relative displacement maps shown as insets in Fig. 2a,b.

**Table 1:** Baseline parameters and finite co-seismic displacements for near-field GNSS and far-field GPS.

		Near-field low-cost GNSS		Far-field GPS^9^		
Baseline	Footwall receiver	MV01 (42.86695°, 13.22725°)	MV03 (42.81554°, 13.25764°)	VETT (42.82450°, 13.27500°)		
	Hangingwall receiver	MV02 (42.86436°, 13.21248°)	MV04 (42.80825°, 13.23962°)	RIFP (42.76270°, 13.17640°)	MSAN (42.76110°, 13.15420°)	ARQT (42.75497°, 13.19873°)
	Total distance (m)	1,286	1,870	10,641	12,230	10,939
	Lateral distance (m)	1,241	1,681	10,591	12,134	10,905
	Vertical distance (m)	−338	−818	−1,024	−1,530	−865
	Azimuth (deg)	267	241	231	236	216
Finite co-seismic displacement: hangingwall relative to footwall	East-West (m)	−0.11	−0.62	−0.46	−0.55	—
	North-South (m)	−0.22	−0.17	−0.27	−0.31	—
	Up-Down (m)	−0.46	−0.81	−0.46	−0.30	−0.50
	Lateral (m)	0.25	0.65	0.53	0.63	—
	Total (m)	0.52	1.04	0.70	0.70	—
	Slip vector azimuth (deg)	206	255	240	241	—

Data from both GNSS pairs show an absence of pre-seismic deformation, as the hangingwall is stationary relative to the footwall for the time preceding the sudden onset of co-seismic displacement. The initiation of relative displacement occurs between 06:40:20 and 06:40:21 for baseline MV01-MV02 and 06:40:21 and 06:40:22 for baseline MV03-MV04. The data then indicate rapid south-westward and downward displacement of the hangingwall relative to the footwall over the following 2–4 seconds. We infer that the data recorded within this period reflect a composite signal of increasing co-seismic displacement together with superimposed high frequency ground motions associated with differential shaking. We estimate rise time (the duration between onset and cessation of co-seismic fault displacement) to be ca. 2–4 s for both baselines assuming that co-seismic displacement on the fault surface ended when all three components of displacement reached their finite offset values (Fig. 2). The 1 Hz temporal frequency of the displacement record effectively limits the resoultion of this approximation to integer seconds. Estimated slip velocities are 0.13–0.26 ms^−1^ and 0.26–0.52 ms^−1^ for baselines MV01-MV02 and MV03-MV04 respectively. Following this short period of large, rapid displacement, the data then indicate only very slight relative movement for the remaining 35–37 seconds. We interpret small fluctuations in displacement during this latter period as the remaining effects of differential ground shaking, as they do not produce further finite displacement.

Table 1 summarises our interpreted values of finite displacement during this event from the two hangingwall-footwall pairs. The finite displacement observed at baseline MV03-MV04 close to the along-strike centre of the rupture trace exceeds that observed along baseline MV01-MV02 located towards the north-eastern tip of the rupture trace. Furthermore, a footwall fault splay behind site MV01 ruptured during the October 30th earthquake. This rupture was not visited on foot, but could be seen from across the valley, and it likely accommodated a portion of the total slip, which is not represented by the GNSS data on baseline MV01-MV02.

## Comparison of GNSS and other datasets

Co-seismic offsets derived from the regional GPS network^9^ (Fig. 1a) show far-field horizontal displacements across the Mt. Vettore fault of 0.53 m (VETT-RIFP) and 0.63 m (VETT-MSAN), in close agreement with the displacement of 0.65 m observed from our near-field GNSS at a similar along strike position on baseline MV03-MV04. The vertical co-seismic displacements measured in the far-field across the Mt. Vettore fault are 0.50 m (VETT-ARQT), 0.46 m (VETT-RIFP) and 0.30 m (VETT-MSAN), which are less than the corresponding near-field displacement of 0.81 m we recorded with GNSS (MV03-MV04), consistent with the localisation of vertical displacement close to extensional faults. Near-field GNSS data for baseline MV01-MV02, approximately 6 km to the north west are more difficult to reconcile with far-field GPS data, due to an increase in structural complexity in this region, including additional synthetic and antithetic faults that produced surface ruptures to the west and east of our GNSS baseline.

InSAR-derived line of sight displacements (24 August, 2016–02 November, 2016, ALOS-2, descending track), averaged within a 200 meter radius of our GNSS units show relative displacements of −0.42 m and −0.97 m for the two baselines. These compare very closely with our near-field GNSS displacement values of −0.41 m and −1.00 m derived by resolving the GNSS displacements into the InSAR line of sight measurement direction.

We visited the GNSS sites and inspected nearby scarp surfaces of the Mt. Vettore fault on 28–29th October (i.e. prior to the M_w_ 6.6 event), and again in the two days following the 30th October earthquake. Our field observations four hours after the earthquake confirmed finite surface rupture displacements had intersected both GNSS baselines (Fig. 1b,c) and that significant slip had occurred on an antithetic fault system to the west of MV02 (Fig. 1d). The measured total displacement of ruptures intersecting baseline MV03-MV04 are similar to the GNSS displacements (−1.21 m downward and 0.44 m horizontal), while those rupture displacements intersecting baseline MV01-MV02 were far less than the corresponding GNSS displacements (−0.06 downward and 0.05 horizontal). These two comparisons are highly informative as they provide a unique insight into two typical situations encountered when comparing near-field displacements to finite surface slip. Firstly, the case of baseline MV03-MV04 where the tectonic system is relatively simple and a good agreement exists between near-field displacement and finite surface displacement. Secondly, the case of MV01-MV02, where the tectonic system is complex and disparities exist between near-field displacement and finite surface displacement that in this case is due to the presence of multiple closely spaced faults with differing orientations and slip vector azimuths.

## Timing of ground rupture

Data from the Italian National Seismic Network^5^ record the hypocentre origin time for the 30^th^ October earthquake as 06:40:17.36 ±0.02 UTC. The accelerometer IT.CLO at Castelluccio village (Fig. 1), installed as part of the Italian Strong Motion Network^15^, recorded an arrival time of 06:40:20.24 UTC, with a duration of 8.86–9.71 seconds^16^ (Fig. 2c). The initiation of acceleration at IT.CLO preceded the rupture-induced co-seismic displacement at our GNSS baselines by no more than 1–2 s. At IT.CLO the peak ground acceleration in the north-south and up-down components occurred at approximately 06:40:23.50, and the east-west component is also close to its maximum value at that time which is near-synchronous with the interpreted cessation of co-seismic displacement at each of our baselines (Fig. 2c).

By comparing the timing of key events across different datasets, we can make the following observations on the evolution of the co-seismic sequence. Surface and near-surface rupture initiated almost immediately (1–2 s) after the onset of shaking at the Castelluccio accelerometer IT.CLO, and significant rupture ceased within five seconds or less after the onset of shaking at IT.CLO. Most, if not all, of the GNSS measured co-seismic displacement occurred prior to the time of peak ground acceleration. Finite displacement across both GNSS baselines was achieved rapidly, within ca. six to eight seconds of the hypocentre origin time (Fig. 2c), demonstrating that the observed surface ruptures are the result of dynamic earthquake slip.

The timing and magnitude of the co-seismic displacements measured using GNSS in the near-field show that the observed surface ruptures are part of a capable fault array on which the surface rupture developed as a direct and immediate response to the propagation of slip from depth during the 30th October earthquake. The implication of this unique observation is that measurements of surface slip obtained through earthquake geology and palaeoseismology can reliably inform physical models of earthquakes where rupture mechanics are required and to aid interpretation of the palaeoseismic record. Alternative mechanisms for the partial or complete generation of some of the surface displacements of this earthquake sequence have been proposed, involving shaking induced shallow landslides and deep seated gravitational slope deformation on a large scale^17–21^. Such mechanisms are very unlikely to be the cause of the displacements we have observed, as landslides and large-scale slope deformation are known to occur progressively over typical time periods of 45–220 seconds^22, 23^, whereas we have shown that finite co-seismic displacement in the near-field occurred rapidly, within six to eight seconds of the hypocentre origin time.

The use of low-cost GNSS receivers located in close proximity to capable faults can significantly enhance our understanding of co-seismic processes and are complementary to existing far-field networks. There is additional scope for further technical development and improved deployment of GNSS receivers, and we are currently testing a number of future enhancements. To provide greater insight into earthquake propagation and surface rupture processes we need to increase the temporal resolution of co-seismic measurements, and are already now using a higher GNSS recording frequency of 10 Hz in other active tectonics projects. Similarly, we are improving spatial resolution by deploying a larger number of receivers in closer proximity to recent surface ruptures, to help investigate spatial variability in near-field displacements during future earthquakes. Remotely operated units that require minimal maintenance will significantly reduce deployment costs.

## Methods

The low-cost GNSS units consist of a single frequency Ublox NEO-M8T GNSS receiver chip with a patch antenna and a 4 GB data logger using universal asynchronous receiver/transmitter (UART). The GNSS chip is configured to output observation data packets of approximately 600 bytes, consisting of code pseudorange, carrier phase, Doppler and signal-to-noise ratios to the data logger at 1 Hz. The packet size is dependent on the number of satellites currently in view at each receiver and the GNSS constellations enabled, however typical data rates at 1 Hz are of the order of 50 MB/day. Each unit is rated at approximately 0.7 W and is powered by a 12 volt 55 Ah lead acid battery which provides power for approximately 40 days. There is no ‘earthquake detection’ as part of the system design; the units log data continuously until power is depleted or the memory of the logger is full. Hence this configuration requires no maintenance or interaction duration operation other than to change the battery and to retrieve data from the data logger. Additional batteries can be installed in parallel to lengthen this maintenance interval. We are developing future versions of these units which will record dual frequency observation data at 10 Hz and be entirely self-sufficient in terms of power and data transfer.

The units were already installed prior to the 30^th^ October earthquake as part of a longer-term project to monitor seismicity following the 24th August Amatrice earthquake. Following retrieval of data from the GNSS units the recorded observation data from each hangingwall receiver and its footwall counterpart was post-processed using static relative positioning to calculate the position of the hangingwall receiver relative to its footwall counterpart with time. The initial position of the footwall receiver of each footwall-hangingwall pair was calculated using Precise Point Positioning (PPP). The recorded observation data were supplemented with navigation, ionospheric, precise orbit and clock data from the International GNSS service (www.igs.org).

## Data Availability

The datasets generated during and/or analysed during the current study are available from the corresponding author on reasonable request.

## Electronic supplementary material

Supplementary Information
