# Supplementary material for: Near-field fault slip of the 2016 Vettore Mw 6.6 earthquake (Central Italy) measured using low-cost GNSS
Source: Sci Rep. 2017 Jul 4;7:4612. doi: 10.1038/s41598-017-04917-w (PMC5496879; doi:10.1038/s41598-017-04917-w)
Supplement: Supplementary file 1 — Supplementary Information [file 41598_2017_4917_MOESM1_ESM.pdf]

Near-field fault slip of the 2016 Vettore M_w 6.6 earthquake (Central Italy) measured using low-cost GNSS

Maxwell W. Wilkinson[1*], Ken J.W. McCaffrey[2], Richard R. Jones[1], Gerald P. Roberts[3], Robert E. Holdsworth[2], Laura C. Gregory[4], Richard J. Walters[5], Luke Wedmore[4], Huw Goodall[4], Francesco Iezzi[3].

1. Geospatial Research Ltd, Department of Earth Sciences, Durham University, DH1 3LE, UK

2. Department of Earth Sciences, Durham University, DH1 3LE, UK

3. Department of Earth and Planetary Sciences, Birkbeck, University of London, London, WC1E 7HX, UK

4. School of Earth and Environment, University of Leeds, Leeds, LS2 9JT, UK

5. COMET, Department of Earth Sciences, Durham University, DH1 3LE, UK

* Correspondence to maxwell.wilkinson@durham.ac.uk

Supplementary information

Baseline	MV01-MV02				MV03-MV04			
Displacement (m)	Horizontal		Vertical	Lateral	Horizontal		Vertical	Lateral
Time (UTC)	E-W	N-S	U-D	Horizontal	E-W	N-S	U-D	Horizontal
06:40:00	0.000	0.000	0.000	0.000	0.000	0.000	0.000	0.000
06:40:01	0.000	0.000	-0.004	0.000	0.001	0.002	0.002	0.003
06:40:02	-0.001	0.000	-0.005	0.001	0.003	0.003	0.002	0.004
06:40:03	-0.001	0.000	0.000	0.001	0.003	0.001	0.006	0.003
06:40:04	0.001	0.000	-0.002	0.001	0.005	0.005	0.001	0.007
06:40:05	-0.002	-0.001	-0.001	0.002	0.002	0.001	0.000	0.002
06:40:06	-0.003	0.000	-0.003	0.003	0.002	0.000	0.002	0.002
06:40:07	-0.001	-0.002	-0.006	0.002	0.001	0.002	0.001	0.002
06:40:08	-0.002	-0.001	0.003	0.003	0.004	0.000	0.003	0.004
06:40:09	0.000	0.000	-0.004	0.000	0.002	0.003	0.001	0.003
06:40:10	0.000	0.001	-0.006	0.001	0.004	0.001	0.006	0.004
06:40:11	-0.001	-0.001	0.004	0.002	0.002	0.001	0.001	0.002
06:40:12	0.000	0.000	0.001	0.001	0.005	0.002	0.002	0.005
06:40:13	-0.003	0.000	-0.002	0.003	0.002	0.001	0.001	0.002
06:40:14	0.000	0.003	-0.001	0.003	0.002	0.003	0.001	0.004
06:40:15	0.000	0.001	0.002	0.001	0.002	-0.001	0.006	0.003
06:40:16	-0.002	-0.003	0.001	0.004	0.002	0.002	-0.001	0.003
06:40:17	0.001	0.000	-0.002	0.001	0.002	0.001	0.000	0.002
06:40:18	0.001	-0.001	0.000	0.002	0.000	-0.001	0.004	0.001
06:40:19	-0.001	-0.002	0.001	0.002	0.001	0.003	-0.002	0.003
06:40:20	0.000	0.002	-0.005	0.002	0.005	0.004	-0.003	0.006
06:40:21	-0.011	-0.001	0.000	0.011	0.002	0.001	0.000	0.002
06:40:22	-0.131	-0.098	-0.296	0.164	-0.026	-0.008	-0.003	0.027
06:40:23	-0.021	-0.172	-0.500	0.174	-0.299	0.150	-0.233	0.335
06:40:24	-0.212	-0.293	-0.452	0.361	-0.696	-0.064	-0.877	0.699
06:40:25	-0.122	-0.188	-0.499	0.224	-0.675	-0.222	-0.686	0.711
06:40:26	-0.087	-0.238	-0.435	0.254	-0.706	-0.137	-0.823	0.719
06:40:27	-0.090	-0.218	-0.411	0.236	-0.603	-0.170	-0.800	0.626
06:40:28	-0.102	-0.238	-0.465	0.259	-0.629	-0.185	-0.825	0.656
06:40:29	-0.099	-0.201	-0.449	0.223	-0.611	-0.174	-0.772	0.635
06:40:30	-0.114	-0.243	-0.453	0.268	-0.642	-0.177	-0.823	0.666
06:40:31	-0.113	-0.234	-0.455	0.260	-0.635	-0.168	-0.805	0.657
06:40:32	-0.105	-0.243	-0.453	0.265	-0.630	-0.178	-0.800	0.655
06:40:33	-0.113	-0.226	-0.449	0.252	-0.627	-0.198	-0.798	0.657
06:40:34	-0.114	-0.233	-0.459	0.260	-0.618	-0.159	-0.803	0.639
06:40:35	-0.118	-0.235	-0.452	0.263	-0.616	-0.173	-0.815	0.640
06:40:36	-0.107	-0.224	-0.465	0.248	-0.623	-0.164	-0.801	0.644
06:40:37	-0.107	-0.229	-0.452	0.253	-0.619	-0.175	-0.811	0.643
06:40:38	-0.106	-0.224	-0.454	0.248	-0.621	-0.162	-0.801	0.642
06:40:39	-0.115	-0.226	-0.454	0.253	-0.622	-0.173	-0.801	0.646
06:40:40	-0.118	-0.232	-0.455	0.260	-0.635	-0.174	-0.809	0.659
06:40:41	-0.105	-0.222	-0.447	0.246	-0.608	-0.165	-0.810	0.630
06:40:42	-0.110	-0.220	-0.458	0.246	-0.625	-0.168	-0.808	0.647
06:40:43	-0.105	-0.226	-0.462	0.249	-0.626	-0.172	-0.808	0.650
06:40:44	-0.110	-0.217	-0.451	0.243	-0.614	-0.175	-0.801	0.639
06:40:45	-0.110	-0.217	-0.459	0.244	-0.622	-0.160	-0.803	0.643
06:40:46	-0.108	-0.221	-0.465	0.246	-0.620	-0.173	-0.805	0.644
06:40:47	-0.112	-0.208	-0.421	0.236	-0.618	-0.172	-0.808	0.642
06:40:48	-0.108	-0.222	-0.466	0.246	-0.628	-0.172	-0.806	0.651
06:40:49	-0.112	-0.224	-0.451	0.250	-0.624	-0.169	-0.807	0.646
06:40:50	-0.112	-0.222	-0.456	0.249	-0.622	-0.172	-0.808	0.645
06:40:51	-0.114	-0.224	-0.470	0.251	-0.619	-0.170	-0.805	0.642
06:40:52	-0.112	-0.207	-0.423	0.235	-0.624	-0.173	-0.806	0.647

06:40:53	-0.100	-0.195	-0.422	0.220	-0.626	-0.174	-0.808	0.650
06:40:54	-0.109	-0.220	-0.465	0.245	-0.620	-0.170	-0.808	0.643
06:40:55	-0.110	-0.219	-0.479	0.245	-0.619	-0.171	-0.806	0.642
06:40:56	-0.105	-0.217	-0.462	0.241	-0.624	-0.172	-0.800	0.647
06:40:57	-0.109	-0.221	-0.467	0.247	-0.625	-0.174	-0.807	0.649
06:40:58	-0.105	-0.202	-0.426	0.228	-0.623	-0.169	-0.814	0.645
06:40:59	-0.109	-0.201	-0.421	0.228	-0.620	-0.170	-0.806	0.642
06:41:00	-0.109	-0.206	-0.417	0.234	-0.625	-0.170	-0.805	0.647

Table S1: Displacement with time of low-cost GNSS baselines MV01-MV02 and MV03-MV04.
